# Cannabis Vaping–Induced Acute Pulmonary Toxicity: Case Series and Review of Literature

**DOI:** 10.1177/2324709620947267

**Published:** 2020-08-05

**Authors:** Sreedhar Adapa, Vijay Gayam, Venu Madhav Konala, Srinadh Annangi, Mina P. Raju, Vishnu Bezwada, Christine McMillan, Hussain Dalal, Amrendra Mandal, Srikanth Naramala

**Affiliations:** 1 Adventist Medical Center, Hanford, CA, USA; 2 Interfaith Medical Center, Brooklyn, NY, USA; 3 Ashland Bellefonte Cancer Center, Ashland, KY, USA; 4 University of Kentucky, Lexington, KY, USA; 5 Ozark Valley Medical Clinic, Ozark, MO, USA

**Keywords:** cannabis, cannabidiol, tetrahydrocannabinol, terpenes, acute respiratory failure, acute respiratory distress syndrome, cannabinoid hyperemesis syndrome

## Abstract

The use of cannabis for recreational as well as medicinal use is on the rise recently with more states legalizing it. We conducted a review analysis of the literature published on acute respiratory failure from vaping cannabis oil. We have also summarized the clinical details (age, length of stay, mode of ventilation, common clinical findings, and steroid use) along with common laboratory abnormalities. This article aims to educate health care providers on the clinical manifestations and management strategies for vaping-induced acute respiratory failure. We also discussed the different available formulations of cannabis oil and key ingredients responsible for the vaping-associated lung injury.

## Introduction

The recreational use of cannabis is increasingly prevalent in the United States, with more states legalizing its recreational and medicinal use. While cannabis has over a 100 active ingredients, most attention is drawn toward 2 main ingredients: cannabidiol (CBD) and tetrahydrocannabinol (THC). THC is mainly responsible for the psychoactive symptoms, while CBD is increasingly used to treat chronic medical comorbidities. CBD is widely available for public usage in various forms as no current regulations exist to dictate usage as it does not cause any psychoactive symptoms. One form, which has picked up significantly, especially among teenagers and millennials is the vaping of cannabis. Vaping cannabis oil is convenient; it is discrete, affordable, and delivers the most amount of the compound compared with other routes of cannabis oil use. We, in this case series, shed light on recent data collected from 7 cases who presented with vaping-associated lung injury (VALI) after vaping cannabis oil.

## Case 1

A 27-year-old Hispanic female college student with no significant past medical history presented to the emergency room with fever, chills, productive cough with dark phlegm, rhinorrhea, headaches along with abdominal pain, and shortness of breath for 2 days. Vital signs revealed fever of 103 °F, heart rate (HR) of 75 beats per minute, respiratory rate (RR) of 16 breaths per minute, blood pressure (BP) 128/77 mm Hg, and saturating 94% on room air. Physical examination was unremarkable except for diminished breath sounds with rales bilaterally. Chest X-ray (CXR) revealed right lung base infiltrate. The patient was diagnosed with community-acquired pneumonia and was discharged on oral azithromycin, ondansetron, and ibuprofen.

**Figure 1.**
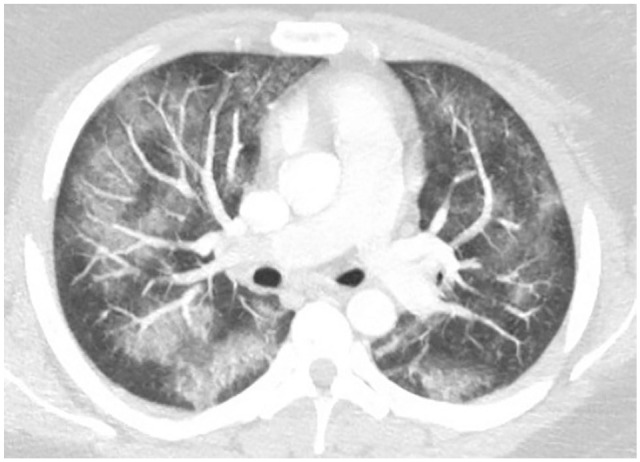
Bilateral patchy ground glass opacities in bronchovascular distribution.

She returned to the emergency room the following day with progressively worsening shortness of breath. Her RR was 24 breaths per minute and saturating 84% on room air. Computed tomography angiogram (CTA) of the chest with and without contrast revealed bilateral patchy ground glass opacities within the bronchovascular distribution (Figure 1). The patient’s clinical condition deteriorated over the next 12 hours requiring high-flow oxygen and repeat imaging showed worsening bilateral pulmonary opacities. On further questioning, the patient admits to smoking marijuana, which she switched to vaping with CBD/THC oil 2 months prior to this presentation, which she bought off the street. She never smoked cigarettes. She denied having any pets, recent travel history, or exposure to any sick contacts. Laboratory workup was significant for leukocytosis, elevated erythrocyte sedimentation rate (ESR), C-reactive protein (CRP), procalcitonin, and urine drug screen was positive for cannabinoids/THC (Table 1). Airway examination was normal on bronchoscopy and bronchoalveolar lavage (BAL) gram stain showed mixed respiratory flora and cultures were negative bacterial, fungal, and viral pathogens. BAL differential showed 5% neutrophils, 10% lymphocytes, and 85% macrophages. Antibiotics were escalated to ceftriaxone and azithromycin for 5 days. She also received methylprednisone 60 mg daily × 3 days, then changed to oral prednisone 40 mg daily with a tapering course of 10 mg per week over a 4-week period. A presumptive diagnosis of VALI secondary to vaping CBD/THC was made as the patient responded very well to the steroids. Her symptoms significantly improved and was discharged home 7 days later without the need for supplemental oxygen.

**Table 1.** Case Series Analysis Table^a^.

Case No.	Age in years	GI symptoms	Fever	Urine drug screen^b^	ESR	CRP	WBC	Procalcitonin level (reference ≤0.5 ng/mL)	Number of days patients was on steroids during hospitalization	Length of stay
1	27	No	Yes	Cannabinoid/THC	83	27	22 000	2.05	7 days	7 days
2	20	Yes	yes	Cannabinoid/THC	86	37.5	17 200	2.19	5 days	6 days
3	38	No	yes	Cannabinoid/THC	111	NA	8000	1.83	5 days	7 days
4	19	No	yes	Cannabinoid/THC	114	38.3	16 800	0.17	4 days	10 days
5	34	Yes	yes	Cannabinoid/THC and benzodiazepines	107	35	12 100	86.64	10 days	12 days
6	27	No	yes	Cannabinoid/THC	NA	NA	15 000	NA	5 days	8 days
7	27	No	yes	NA	90	NA	16 900	0.46	16 days	16 days

Abbreviations: GI, gastrointestinal; ESR, erythrocyte sedimentation rate (reference value = 0-20 mm/h); CRP, C-reactive protein (reference value ≤0.9 mg/dL); WBC, white blood cell count (reference value = 4400-11 000 mm^3^); THC, tetrahydroxy cannabinoids; NA, not available.

a The following infectious and autoimmune workup is performed in all 7 cases and resulted negative: BIOFIRE FILMARRAY respiratory panel 2, serum *Mycoplasma* Ab, serum coccidioidomycosis Ab, serum *Chlamydia trachomatis* AB Titer TB QuantiFERON gold, HIV1/2 Ab/p24 Ag, urine *Streptococcus* and *Legionella* antigen, serum *Cryptococcus* antigen screen, *Aspergillus* antibody, *Mycoplasma pneumonia* by polymerase chain reaction, hypersensitivity pneumonitis panel (*Aspergillus fumigatus* #1, *Aspergillus fumigatus* #2, *Aspergillus fumigatus* #3, *Aspergillus fumigatus* #6, *Aureobasidium pullulans, Pigeon Serum*, *Micropolyspora faeni, Thermoactinomyces vulgaris* #1, *Aspergillus flavus* #2, *Saccharomonospora viridis*, and *Thermoactinomyces candidu*), autoimmune testing panel (rheumatoid factor, cyclic citrullinated peptide, antineutrophilic cytoplasmic antibody, myeloperoxidase antibody, proteinase 3 antibody, antinuclear antibody, anti-RO antibody, anti-LA antibody, Scl-70, anti-Jo1, creatinine phosphokinase, immunoglobulin E levels).

b Urine drug screen tests for amphetamines, methamphetamines, barbiturates, benzodiazepines, cannabinoids/THC, cocaine, methadone, opiates, oxycodone, phencyclidine, and tricyclics.

## Case 2

**Figure 2.**
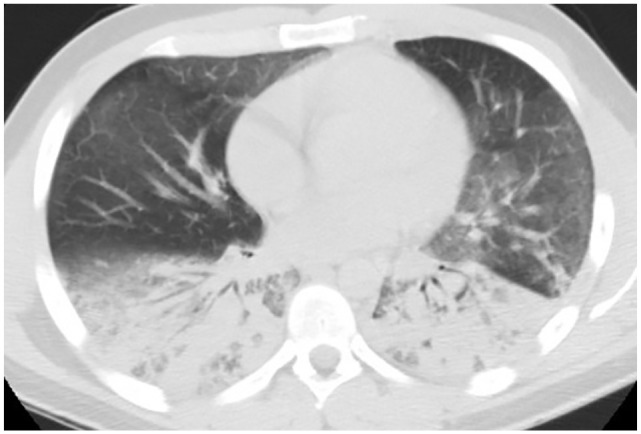
Mild ground glass opacities to upper lobe with bilateral lower lobe dense consolidations.

A 20-year-old Caucasian male college student with medical history of depression and anxiety presented with subjective fevers, shortness of breath, productive cough, nausea, vomiting, and epigastric pain for 4 days. Patient admits experiencing similar symptoms 2 months prior to this presentation and was treated with oral antibiotics for 10 days as an outpatient. He denied smoking cigarettes but admits vaping cannabinoid (CBD/THC mix) oil. Denies having any pets, recent travel, or exposure to sick contacts. Vital signs on presentation were temperature of 103 °F, HR 111 beats per minute, BP 130/83 mm Hg, RR 40 breaths per minute, and saturating 91% on room air. Physical examination was significant for respiratory distress and decreased breath sounds on both lung bases. Laboratory workup was significant for leukocytosis, elevated ESR, CRP, procalcitonin, and urine drug screen was positive for cannabinoids/THC (Table 1). Urine drug screen was positive for cannabinoids/THC. Extensive infectious workup including sputum studies, urine, and serum studies were negative (Table 1). CXR showed bilateral air space disease and CT of the chest without contrast showed bilateral upper lobe ground glass opacities and lower lobe dense consolidations (Figure 2). Ultrasound of the abdomen was unremarkable. Supportive care was provided by oxygen supplementation via high-flow nasal cannula to maintain saturations above 90%. He was initiated on methylprednisolone 60 mg intravenously (IV) twice a day for presumed VALI. He was discharged home with 2 L of supplemental oxygen via nasal cannula on prednisone 40 mg daily with a 3-week steroid taper regimen.

## Case 3

**Figure 3.**
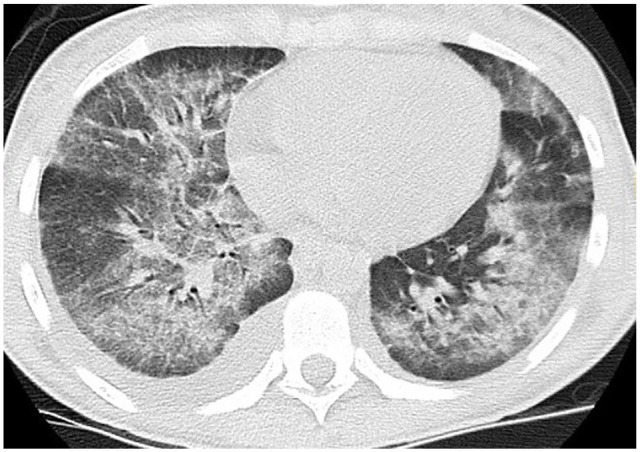
Bilateral ground glass patchy infiltrates with subpleural sparing and small right pleural effusion.

A 38-year-old old Hispanic male with medical history of anxiety presented to the emergency room complaining of fever, shortness of breath, productive cough with green phlegm, and body aches for 2-day duration. He denies any sick contacts or recent travel. He admits to vaping CBD/THC, most recently purchased from a local Los Angeles county dispensary. Vitals signs on presentation were temperature 102.5 °F, HR 113 beats per minute, BP 132/82 mm Hg, RR 24 breathes per minute, and oxygen saturation of 87% on room air. Physical examination was remarkable for respiratory distress and bilateral coarse breath sounds on auscultation. Laboratory workup was significant for elevated ESR, procalcitonin, and urine drug screen was positive for cannabinoids/THC (Table 1). Initial CXR showed bilateral infiltrates and CT chest without contrast showed bilateral patchy glass patchy opacities with subpleural sparing and small right pleural effusion (Figure 3). Extensive infectious workup including sputum studies, urine, and serum studies were negative (Table 1). The patient was subsequently diagnosed with respiratory failure secondary to VALI from CBD/THC vaping. He was started on IV methylprednisone 60 mg daily for 2 days and then converted to oral prednisone 40 mg daily. He clinically improved and was discharged on room air with steroid taper to be completed in 2 weeks.

## Case 4

**Figure 4.**
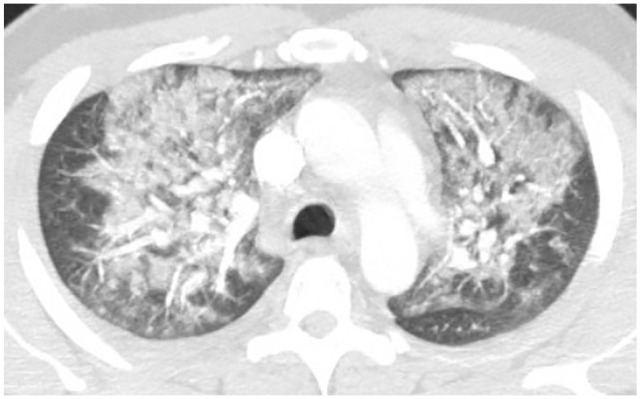
Bilateral upper lobe ground glass opacities centrally distributed.

A 19-year-old Hispanic female with no significant past medical history presented with fever, chills, shortness of breath, and productive cough with whitish phlegm for 4 days. Her social history was significant for vaping-flavored nicotine and vaping CBD/THC oil 1 week prior to developing current symptoms. She also admits decreased appetite with a 15-pound weight loss since the last month. Denies any recent travel history or exposure to sick contacts. Vital signs on presentation were temperature of 100.7 °F, HR 137 beats per minute, BP 105/60 mm Hg, RR 18 breaths per minute, and oxygen saturation of 98% on 2 L nasal cannula. Lungs were clear to auscultation and the rest of the physical examination was also unremarkable. Laboratory workup was significant for leukocytosis, elevated ESR, CRP, and urine drug screen positive for cannabinoids/THC (Table 1). Initial CXR showed left basilar infiltrate and she was started on levofloxacin for presumed pneumonia. Her respiratory status declined over the next few days and CTA of chest later showed bilateral upper lobe ground glass opacities (Figure 4). Airway examination was normal on bronchoscopy and BAL gram stain showed mixed respiratory flora and cultures were negative for bacterial, fungal, and viral pathogens. BAL differential showed 60% neutrophils, 30% lymphocytes, and 10% macrophages. Given negative infectious workup, no other etiology identified for respiratory failure and imaging consistent with VALI from CBD/THC vaping, she was initiated on 60 mg of IV methyl prednisone daily and switched to 40 mg of oral prednisone 2 days later. Her symptoms subsequently resolved, and she was sent home on room air with a tapering steroid course over 2 weeks.

## Case 5

**Figure 5.**
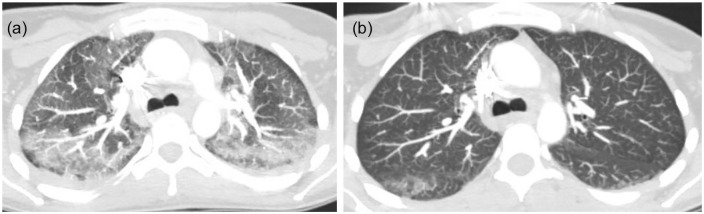
(a) Pretreatment—bilateral upper lobe patchy ground-glass infiltrates. (b) Posttreatment—interval improvement of patchy bilateral upper lobe infiltrates with small residual superior segment right lower lobe ground-glass opacity.

A 34-year-old Caucasian female waitress at a local restaurant presented with complaints of worsening dyspnea, productive cough with yellow phlegm, nausea, vomiting, generalized weakness, and diarrhea for 2 weeks duration. She was prescribed azithromycin 10 days prior to this presentation without any improvement in her symptoms. She denied any sick contacts or travel abroad. She is a current cigarette smoker and also started vaping CBD/THC oil a year ago. She admits vaping CBD/THC oil bought from a local dispensary 2 days prior to her symptom initiation. On physical examination, the patient was in respiratory distress with vital signs of temperature 100.8 °F, HR 136 beats per minute, BP 123/88 mm Hg, RR 22 breaths per minute, and oxygen saturation of 88% on room air. Physical examination was significant for decreased breath sounds throughout the lung fields. Laboratory workup was significant for leukocytosis, elevated ESR, CRP, and urine drug screen was positive for cannabinoids/THC (Table 1). Extensive infectious workup including sputum studies urine and serum studies were negative (Table 1). CXR showed bilateral interstitial infiltrates and CTA of the chest with and without contrast confirmed bilateral upper lobe ground glass patchy infiltrates (Figure 5(a)). She was diagnosed with acute hypoxic respiratory failure likely from VALI secondary to vaping CBD/THC oil. The patient was started on IV methylprednisolone 60 mg daily and then transitioned to oral prednisone 40 mg daily. A repeat CT chest with contrast 6 days after steroid initiation showed interval improvement of patchy bilateral infiltrates with a small residual superior segment of right lower lobe ground glass opacity (Figure 5(b)). She was discharged home with a tapering course of steroids for a total to 2 weeks.

## Case 6

**Figure 6.**
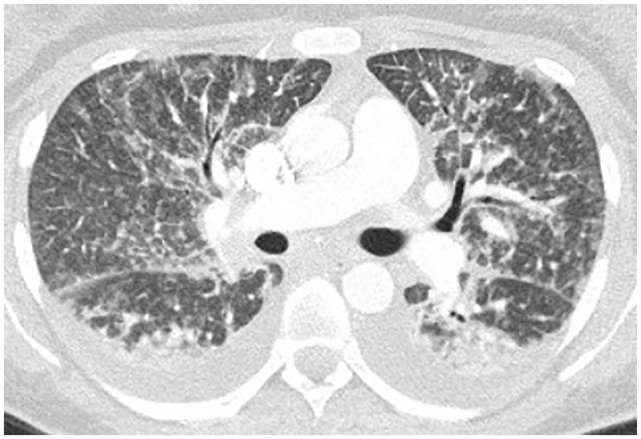
Bilateral pleural effusions with peribronchovascular ground glass infiltrates with septal thickening, nodular pattern.

A 27-year-old Hispanic female with no significant past medical history presented with progressively worsening dyspnea, nonproductive cough, subjective fevers, nausea, decreased appetite, and fatigue for 10 days. The patient was also vaping CBD/THC oil intermittently. She was alert and oriented on physical examination and in mild respiratory distress, noted to have diffuse bilateral wheezing with rhonchi on auscultation. Vital signs showed temperature 102 °F, HR 122 beats per minute, BP 131/72 mm Hg, RR 24 breaths per minute, and oxygen saturation of 96% on 2 L nasal cannula. Laboratory workup was significant for leukocytosis, elevated ESR, CRP, and urine drug screen was positive for cannabinoids/THC (Table 1). Extensive infectious workup including sputum studies urine and serum studies were negative (Table 1). CTA of the chest showed bilateral diffuse ground glass opacities with septal thickening, tree in bud opacities, and bilateral pleural effusions (Figure 6). She was diagnosed with community-acquired pneumonia and was started on azithromycin and ceftriaxone. As the hypoxia did not improve with antibiotics, she was later started on IV methylprednisolone 60 mg daily for 3 days, changed to oral prednisone 40 mg daily for presumed VALI. Extensive infectious workup including sputum studies urine and serum studies were negative (Table 1). She received azithromycin and ceftriaxone for a total of 5 days during her hospital stay. Repeat CXR showed an improvement, and the patient was discharged home with a steroid taper for a total of 2 weeks.

## Case 7

**Figure 7.**
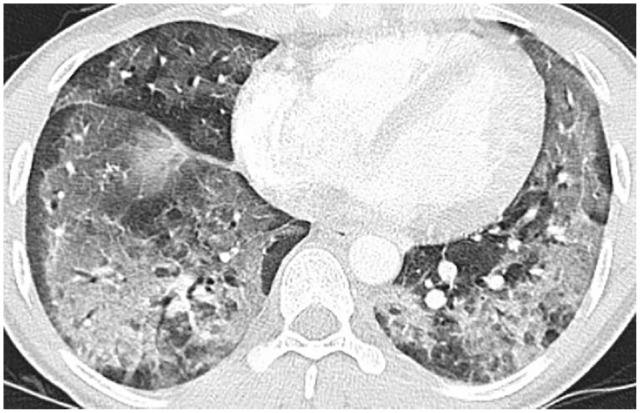
Diffuse bilateral ground glass opacities with crazy paving pattern with subpleural sparing.

A 27-year-old Hispanic male presented to the urgent care with complaints of fatigue and generalized body aches for 2 weeks with fever 101.5 °F. CXR at that time showed basilar interstitial infiltrates and was sent home with doxycycline for presumed pneumonia. Subsequent follow-up with primary care provider 3 days later showed worsening dyspnea and a new left side pleuritic chest pain. The patient was advised to complete his prescribed antibiotic course and also given a prescription for albuterol, fluticasone-salmeterol inhalers. The following day, the patient presented to the emergency room with worsening respiratory symptoms and is concerned that he is having vaping lung injury after seeing a local news report about the possible lung problems associated with vaping. The patient admitted vaping THC daily. He complained of worsening shortness of breath on exertion along with decreased appetite. He denied any nausea and vomiting. On physical examination, the patient was in respiratory distress with temperature of 99.2 °F, HR 128 beats per minute, BP 135/79 mm Hg, RR 31 breaths per minute, and oxygen saturation of 89% on 4 L of nasal cannula. Auscultation of the lungs revealed diminished coarse breath sounds throughout the lung fields. Pertinent abnormal laboratory workup was mentioned in Table 1. CXR showed bilateral patchy opacities and CTA of the chest showed diffuse ground-glass opacities with crazy paving pattern with subpleural sparing (Figure 7). Extensive infectious and autoimmune workup was negative. The patient was diagnosed with VALI secondary to vaping THC. He was started on high dose of methylprednisone 60 mg IV every 12 hours for 2 days then transitioned to oral prednisone. He decompensated after switching to oral steroids, hence IV methylprednisone 60 mg IV q12 hours was restarted for 5 more days, then again tapered to oral steroids. His oxygen saturation was maintained with high-flow nasal cannula at 70% and 50 L. He was discharged home on 2 L of oxygen via nasal cannula along with steroid taper over the next 3 weeks.

## Discussion

*Cannabis sativa* is a flowering plant indigenous to eastern Asia but now is cultivated throughout the world due to its widespread industrial uses.^1-4^ Hemp, or industrial hemp, is one of the strains of the *Cannabis sativa* plant, which has a low concentration of THC and a high concentration of CBD, has a variety of industrial uses.^5-9^ Marijuana is another strain of *Cannabis sativa* but has a higher concentration of THC and a lower concentration of CBD.^5,10-13^ CBD is one of the nonpsychotropic cannabinoid that is extracted from the *Cannabis sativa* plant. THC on the other hand, is the psychoactive cannabinoid, which is labeled as the “high.”^14,15^ CB1 and CB2 are the 2 most common cannabinoid receptors that mediate the effects of CBD and marijuana in humans.^16^ CB1 receptors are mainly located in the central nervous system, whereas CB2 receptors interact with the immune system.^16^ Cannabis has earned its reputation for its uses in a slew of chronic morbidities. Cannabis is known to work on the body’s endocannabinoid system, which is known to regulate functions such as sleep, immune system, and pain perception.^17,18^ Cannabis is shown as an effective antiemetic agent among cancer patients with chemotherapy-induced nausea/vomiting.^19^ Last, CBD has Food Drug Administration approval in the form of a drug named Epidiolex, to treat seizures associated with Lennox-Gastaut syndrome and Dravet syndrome.^20,21^

Cannabinoids are being consumed in varied formulations, and vaping is one such delivery method that has been increasingly used in the recent times.^22^ Vaping is defined as the process of inhaling vapor produced by e-cigarette or a similar device.^23^ Electronic cigarettes or vaping devices are used to deliver nicotine, cannabinoids (CBD/THC), flavoring agents, chemicals, and other substances of interest to the consumers. Vaping device consists of a vape cartridge containing the desired material in the liquid formulation (nicotine or cannabinoids) along with a mouthpiece, battery, and a heating component that converts the liquid formulation in the cartridge to a vapor form facilitating inhalation. Most cannabis vaping cartridges contain a combination of CBD and THC in different individual concentrations. Along with the commonly used CBD and THC, terpene is another compound that gained attention in recent times.^24^ Terpenes are hydrocarbons compounds found naturally in the essential oils of plants, synthesized from trichomes of the plant.^25^ It is presumed that terpenes aid in better absorption of CBD and THC causing an entourage effect.^24^ However, trepenes in the cannabis extract when heated to high temperatures during vaping process degrade into methacrolein and benzene, which are known to cause acute lung injury and pulmonary edema leading to respiratory failure.^26-29^ CBD/THC oil used in the vaping cartridges also contain various harmful compounds, largely depend on the type of extraction solvent used.^30,31^ Commonly used solvents are butane, propane, ethanol, and carbon dioxide. Cannabis extracted using butane oil is called butane hash oil (BHO), contains a very high concentration of THC at 80% compared with only 10% to 25% with other extraction methods.^32-36^ However, smoking BHO that contains butane oil was linked to acute respiratory failure requiring intubation in a 19-year-old male and the proposed mechanism was a direct injury resulted from inhalation of butane and/or other impurities.^37^ In another case reported by Anderson et al, an 18-year-old female was diagnosed with pneumonitis and respiratory failure secondary to using BHO-processed cannabis. Vitamin E acetate is another additive commonly added to THC-based cigarettes or vaping products. Though benign with oral intake, inhalation of vitamin E acetate aerosols was associated with an increase in the markers of lung injury by analyzing bronchoalveolar lavage fluid.^38^ The presence of vitamin E in alveolar lavage fluid of 94% subjects diagnosed with VALI further strengthens its etiological role.^39^ Heating vitamin E to high temperatures during the vaping process also releases toxic ketene gas, directly toxic to the lungs.^40^ Furthermore, chemicals released from vaping of cannabis may also damage the bronchial epithelium and disrupt alveolar surfactant thus interfering with gas exchange leading to respiratory failure.^41^

With the increasing use of cannabis-based products and vaping devices in the United States, vaping cannabis has emerged as a major route of cannabis consumption.^42,43^ Cannabis is sold in cartridges, which are slim disposable electronic cigarette tanks filled with cannabis juice. These cartridges can then be used with vaping devices that are commercially available in the market.^44^ While it has shared the spotlight continuously for all its health benefits and virtually no side effects, little attention has been paid to any significant adverse events associated with cannabis use until recently, where there has been a surge in the number of cannabis-related vaping cases and acute lung injury leading to respiratory failure. The series of cases we presented above suggest a possible etiological link between vaping cannabis and respiratory failure. All our cases vaped a combination of CBD and THC, except case 7, who only vaped THC. However, on further question, our cases and information from local dispensaries, we found terpenoids are added to the CBD/THC extract to enhance the flavor, solvent properties, and to attain the synergistic effect. Whether the noted respiratory failure in our cases is secondary CBD/THC, or terpene or solvent impurities and additional ingredients present in the cartridge is yet to be determined. Along with respiratory distress, our cases also experienced flu-like symptoms, fatigue, fevers, and gastrointestinal symptoms like nausea, vomiting, and abdominal cramps.

A high index of clinical suspicion combined with detailed history, supported by imaging studies, and the absence of an alternative etiology for respiratory failure is the key to diagnose VALI. It should be highly suspected among cases who develop respiratory distress after vaping along with a positive drug screen, consistent laboratory, and imaging findings after ruling out other alternative etiologies including infections. Among cases with VALI, elevated white blood cells count was reported in 87% of cases and ESR >30 mm/h in 93% of cases, both have no predictive value in distinguishing vaping lung injury from infectious etiologies.^45^ Radiographic findings consistent with VALI include bilateral pulmonary infiltrates on CXR and ground glass opacities on CT chest. CXR should be obtained in all suspected cases and CT chest among cases with normal or nondiagnostic CXR findings.^45^ CXR underestimated the extent of lung damage in few of our cases and CT chest should be performed where there is a high index of suspicion. Apart from supportive care by providing oxygen supplementation, initiation of steroids or antibiotics depend on the initial clinical presentation and subsequent clinical course. Routine use of steroids is not advisable, and the decision on steroid initiation is based on individual clinical assessment. While early initiation among cases with severe respiratory distress and withholding steroids among cases with alternative diagnosis is universally accepted, early initiation versus conservative management among less severe cases is still a topic of debate. Recommended dose includes methylprednisolone equivalent of 0.5 to 1 mg/kg/day, tapered over 1 to 2 weeks largely guided by the clinical improvement.^45-49^ While no randomized clinical trials are available to guide the optimal dose and duration of steroids, current recommendations are largely based on case series analysis.^45,50^ While the presence of fever, elevated white blood cells count, and tachycardia in a patient with respiratory distress points toward infectious etiology, their presence do not necessarily rule out VALI. Empiric antibiotics should be initiated in those cases for possible pneumonia awaiting results of infectious workup and clinical improvement.^45,48,49^ Lack of clinical improvement with antibiotics in such cases with consistent exposure history strongly point toward alternative etiology like VALI.

After documenting clinical stability for 1 to 2 days, patients can be safely discharged but a close follow-up is required owing to the increased risk of worsening and readmission among cases with VALI. Patients should also be educated to stay away from vaping strictly. High clinical suspicion along with early diagnosis and appropriate clinical intervention is crucial and has led to the successful recovery in our patients.

## Conclusion

Vaping cannabis oil can lead to acute respiratory failure. A high index of suspicion among treating physicians is needed to facilitate prompt diagnosis and treatment initiation. Our case series and above discussion should help increase physician awareness on the clinical presentation of these cases, to aid in the early diagnosis and prompt management, thus decreasing the morbidity, mortality, and prolonged hospital course.
